# Percutaneous kyphoplasty for the treatment of very severe osteoporotic vertebral compression fractures with spinal canal compromise

**DOI:** 10.1186/s13018-018-0719-z

**Published:** 2018-01-17

**Authors:** Heng Wang, Zongyu Zhang, Yijie Liu, Weimin Jiang

**Affiliations:** 1 Department of Orthopaedic Surgery, The First Affiliated Hospital of Soochow University, 899 Pinghai Road, Suzhou, China; 2 Department of Orthopaedic Surgery, Lianyungang Affiliated Hospital of Nanjing University of Chinese Medicine, 148 Chaoyang Road, Lianyungang, China

**Keywords:** Kyphoplasty, Osteoporosis, Very severe osteoporotic vertebral compression fracture, Spinal canal compromise

## Abstract

**Background:**

Very severe osteoporotic vertebral compression fractures (vsOVCFs) are osteoporotic vertebral compression fractures with vertebral body collapse to less than one third of their original height. Few data are available about the use of percutaneous kyphoplasty (PKP) in treating vsOVCFs with spinal canal compromise. The aim of this study was to evaluate the safety and efficacy of percutaneous kyphoplasty (PKP) for the treatment of vsOVCFs with spinal canal compromise.

**Methods:**

Thirty-five patients who suffered vsOVCFs with spinal canal compromise but without neurological deficits were treated by PKP between January 2009 and October 2014. The vertebral height, local kyphotic angle (LKA), visual analogue scale (VAS) and Oswestry Disability Index (ODI) values were assessed before the operation, 1 day after the operation and at the final follow-up.

**Results:**

Significant improvements on the VAS and ODI were noted 1 day post-operatively (*p* < 0.01), and these results were preserved at the final follow-up. The vertebral height was restored and the LKA was improved after surgery (*p* < 0.01). No neurological deterioration was found. Five of 35 vertebrae (14.3%) of cement leakages were all asymptomatic. Four new OVCFs in three patients were identified.

**Conclusion:**

PKP is a safe and effective procedure for the treatment of vsOVCFs with spinal canal compromise, achieving significant vertebral height restoration and kyphotic angle reduction and leading to a significant pain relief and improvement in function.

## Background

Osteoporotic vertebral compression fracture (OVCF) is a common cause of pain and disability in the elderly population, affecting 1.4 million people each year worldwide [1]. Conservative management, including bed rest, pain relievers, bracing and physical therapy, may fail to relieve pain and frequently lead to prolonged immobilisation, depression and a substantial negative impact on life quality [2–4].

Percutaneous kyphoplasty (PKP) is a minimally invasive procedure for the treatment of OVCFs, and numerous encouraging studies have been reported [2, 5, 6]. However, there have been only few reports on the role of PKP in the treatment of very severe osteoporotic vertebral compression fractures (vsOVCFs), which are the vertebral body collapse to less than one third of their original height [7, 8]. Even less data are available about the use of PKP in treating vsOVCFs with spinal canal compromise.

Here, we report on our experience of the treatment for vsOVCFs with spinal canal compromise by PKP. The purpose of our study was to evaluate the efficacy and safety of PKP for the treatment of vsOVCFs with spinal canal compromise.

## Methods

### Study population

**Fig. 1:**
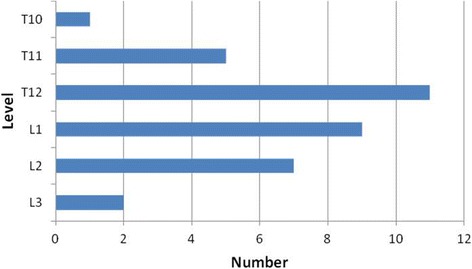
The distribution of levels treated by PKP among 35 patients suffering from vsOVCFs with spinal canal compromise

A total of 35 patients who suffered from vsOVCFs with spinal canal compromise at one level underwent PKP between January 2009 and October 2014. The mean age of the patients was 71.7 years old (65 to 82). The mean duration of symptoms was 5.3 months (1.5 to 10). All the patients had osteoporosis, which was preoperatively identified by dual-energy X-ray absorptiometry (DXA). The mean *T* score was − 4.05 ± 0.76 (− 5.6 to − 3.1) as measured from the L-spine. All the patients had severe back pain which was refractory to conservative therapy, such as bed rest and treatment with analgesics and anti-osteoporotic medication. The pain region was consistent with the presence of oedema in the fractured vertebra found on MRI. Patients were excluded due to the presence of pathologic compression fractures, neurologic deficits and spinal cord compression syndrome. The treated levels were localised in the thoracic spine and the lumbar spine with majority between T12 and L2 (Fig. 1). This study was approved by the Institutional Ethics Committee of Soochow University.

### Surgical technique

All PKP procedures were performed under general anaesthesia using fluoroscopic guidance. Patients were placed in the prone position with a bolster placed under the sternum and pelvis. Guide wires were inserted in order to obtain bilateral transpedicular access to the fractured vertebral body. The opening was gradually enlarged using successively larger cannula. Kyphon balloon tamps (Kyphon Inc., Sunnyvale, CA, USA) were inserted through the working channel and placed in the anterior three fourths of the vertebral body in the lateral view. The balloons were then inflated slowly to reduce the fracture and to create a cavity for the injection of cement. The inflation was stopped when the pressure reached 200 psi or the balloon contacted the endplate. They were then deflated and removed. Polymethylmethacrylate (PMMA) cement was injected incrementally to fill the cavity when it became doughy and could stand at the tip of the bone cement inserter. The entire injection process was monitored continuously under fluoroscopic control in the lateral view. The procedure was stopped immediately if high resistance was encountered or if PMMA neared the posterior wall of the vertebral body. The amount of cement was noted. The patients were discharged 1 day after the procedure and were referred for anti-osteoporosis treatment in the outpatient department.

### Data collection and outcome assessment

We recorded clinical and radiological evaluations at pre-operation, post-operation (1 day after surgery) and final follow-up.

Visual analogue scale (VAS; scored from 0 to 10: 0, no pain; 10, the worst imagined) was used to evaluate the pain, and Oswestry Disability Index (ODI) score was used as a functional assessment.

The anterior and middle heights of the vertebra were measured on standing lateral radiographs. The normal height of the fractured vertebra was estimated from the mean of the measurements from the closest normal vertebra cephalad and caudad to the treated level. The vertebral height ratios were calculated as follows: (fractured vertebral height/mean adjacent control vertebral height) × 100% [9]. Local kyphotic angle (LKA) was calculated using the Cobb method: the angle formed between a line drawn parallel to the superior endplate of the vertebra one level above the fracture and a line drawn parallel to the inferior endplate of the vertebra one level below the fracture.

### Statistical analysis

Data were presented as mean ± SD. The Statistical Package for the Social Sciences software (version 16.0, SPSS, Chicago, IL, USA) was used for the analysis. Comparisons of clinical and radiological outcomes pre- and post-operatively were made using a paired *t* test. Differences were considered statistically significant when *p* < 0.05.

## Results

All 35 patients tolerated the operation well. No neurological deterioration was found. The average operative time was 41.3 ± 5.4 (32–51) min. The average cement volume was 4.95 ± 0.68 (3.5–6.0) ml. The mean follow-up was 34.0 ± 8.1 (18–48) months.

All patients achieved substantial pain relief after surgery. The VAS score decreased significantly from a pre-operative value of 8.3 ± 1.1 to a post-operative value of 2.6 ± 0.8 and was maintained at 2.8 ± 0.8 at the final follow-up. The ODI score decreased significantly from a pre-operative value of 76.6 ± 12.9 to a post-operative value of 34.3 ± 7.7 and was 35.5 ± 9.3 at the final follow-up (Table 1).

**Table 1:** Mean improvement in VAS and ODI

	Pre-operative	Post-operative	Last follow-up
VAS	8.3 ± 1.1	2.6 ± 0.8^a^	2.8 ± 0.8^a^
ODI (%)	76.6 ± 12.9	34.3 ± 7.7^a^	35.5 ± 9.3^a^

*VAS* visual analogue scale, *ODI* Oswestry Disability Index

^a^*P* < 0.01 compared to pre-operative value

**Fig.2:**
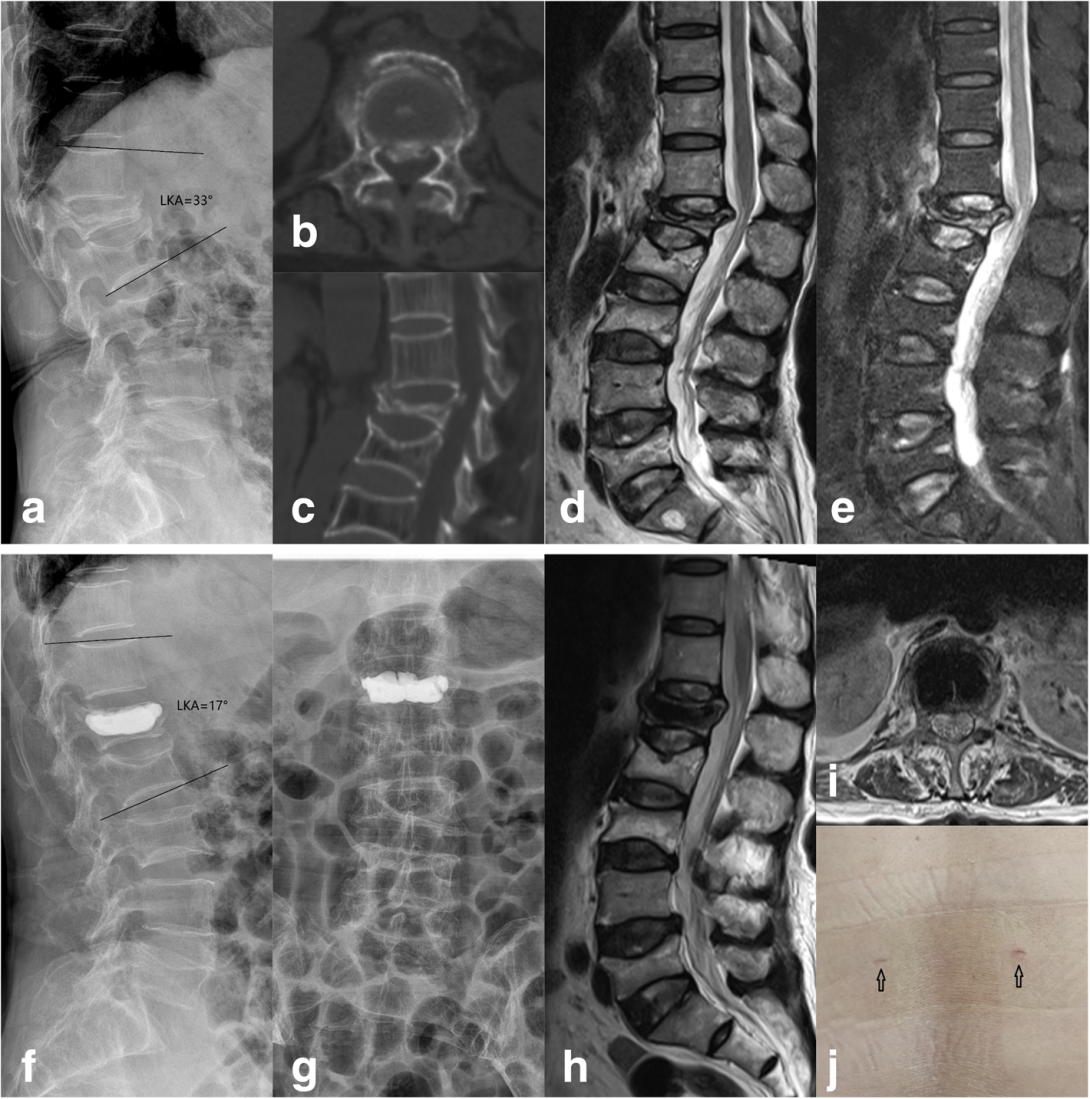
A 69-year-old woman suffered L1 vsOVCF with spinal canal compromise treated with percutaneous kyphoplasty. **a** The pre-operative kyphotic angle was 33°. **b**, **c** Pre-operative CT showed retropulsion of the posterior wall fragment into the spinal canal. **d**, **e** The pre-operative MRI displayed high signal intensity in the vertebral body in T2-weighted and short tau inversion recovery (STIR) images. **f**, **g** The post-operative X-ray films showed cement distributed well and did not leak. The kyphotic angle was 17°. **h**, **i** The post-operative MRI showed reduction of the retropulsed bone fragment from the spinal canal. **j** The minimal incisions of PKP (black arrows)

Significant increases of the anterior and middle vertebral heights were observed after surgery, and the vertebral heights maintained throughout the period of follow-up. The mean improvement in LKA was 9.3°, and the correction was maintained at the final follow-up (Fig. 2, Table 2).

**Table 2:** Mean improvement of radiographic data

	Pre-operative	Post-operative	Last follow-up
Vertebral body height ratios (%)^a^			
Anterior	26.5 ± 4.2	53.6 ± 4.0^b^	51.2 ± 3.9^b^
Middle	30.9 ± 4.5	60.9 ± 4.3^b^	57.4 ± 4.2^b^
LKA (°)	25.1 ± 5.0	15.3 ± 5.5^b^	15.7 ± 5.8^b^

*LKA* local kyphotic angle

^a^Vertebral body height ratios (%) = (fractured vertebral body height/normal vertebral body height) × 100%

^b^*P* < 0.01 compared to pre-operative value

Asymptomatic cement leakage occurred in 5 of 35 cases (14.3%) with 2 cases into the intervertebral space, 2 cases lateral to the vertebral body and 1 case into the paravertebral vein. During the period of follow-up, three patients had developed four new symptomatic OVCFs and underwent additional PKP to relieve pain. There was no infection, pulmonary embolism and patient death.

## Discussion

The treatment for vsOVCFs with spinal canal compromise but without neurological deficits remains controversial. Conservative treatment does not appear to be effective for painful vsOVCFs, which often results in many complications, such as pulmonary deterioration, deep vein thrombosis, weight loss and depression [3, 10]. Open surgery is also inappropriate in these patients due to severe surgical trauma, difficulty of fixation and long time of bedding [11, 12].

Percutaneous vertebroplasty (PVP) and PKP have been proven to be safe and effective to treat OVCFs time and time again [2, 5, 6, 13–15]. However, in vsOVCFs with spinal compromise, many authors considered these two procedures to be relatively or even absolutely contraindicated because of technical difficulty, high risk of cement leakage and further spinal canal compromise [16–19]. Nevertheless, the literature and our own experience demonstrate this assertion to be unfounded. Some studies reported good results with PVP owing to its analgesic and stabilising effects in the treatment of osteoporotic vertebral fractures with spinal canal compromise [18, 20, 21]. There are also some studies proving that PVP can be used in vsOVCFs with satisfactory results [7, 8, 22]. In our study, the VAS and ODI scores decreased significantly after the procedure, demonstrating that PKP could provide rapid, marked, and sustained improvements in pain and function in the treatment of vsOVCFs with spinal compromise.

In vsOVCFs with spinal canal compromise, needle placement is technically demanding, because of the extreme kyphotic angle and the severe vertebral collapse. In our study, to avoid perforating the endplate, a low lateral transpedicular approach was adopted and bilateral double balloon inflation was performed to achieve en masse reduction [23] in all cases.

Cement extravasation is the most common complication in PKP and PVP. Cement leakage into the spinal canal is devastating, resulting in spinal cord or nerve injury. In vsOVCFs with spinal compromise, the posterior vertebral walls are deficient and other peripheral vertebral walls are frequently damaged as well. Therefore, the risk of cement leakage is very high. Young et al. [22] reported that cement leakage was demonstrated in 72% cases using PVP for vsOVCFs. Nieuwenhuijse et al. [7] reported that cement leakage occurred in 91.9% of vsOVCFs treated by PVP. Li et al. [21] showed that cement leakage rate was 47.8% using PVP for the treatment of osteoporotic vertebral fractures with spinal compromise. In the current study, cement leakage occurred in 5 of 35 treated vertebra (14.3%). The lower cement leakage rate could be ascribed as follows: (1) The cavity created by balloons provided space for bone cement filling under conditions of high viscosity and low pressure. (2) We inflated the balloons slowly and moderately to avoid excessive reduction of the fracture which may cause new defects of the vertebral body. (3) We controlled the cement with a doughy consistency, which did not stick to the finger and could stand erect at the tip of the bone cement syringe when pushed out, to block some cortical defects and segmental veins [24]. (4) Continuous C-arm X-ray monitoring was performed during the entire gradual injection process. Injection was stopped immediately when 1/4 of the distance to the posterior wall of the vertebral body remained. (5) For patients with the anterior wall deficiency, a small amount of mid- to later-stage cement in dough phase was first injected to block the defect of the anterior wall. After the filling solidified, earlier stage cement in dough phase was then injected.

Several studies have demonstrated that, compared to PVP, PKP can effectively correct kyphosis and improve vertebral body height [25, 26]. In vsOVCFs with spinal canal compromise, the severe compression fracture generated a backward force which resulted in bone fragment displacement into the spinal canal. The balloon inflation can distend the vertebral body to partially restore its height, which would counterbalance the backward force. If related ligaments are structurally complete, the distention of the vertebral body can make the ligaments tense to retract protruded bone fragments. In the present study, significant post-operative correction of the kyphosis and restoration of the vertebral height were observed and were not lost during follow-up. No neurological deterioration was found.

The limitations of our study are its retrospective nature and small number of patients. A further limitation is the lack of a control group. Thus, further large-scale prospective randomised controlled studies are required.

## Conclusion

PKP is a safe and effective procedure for the treatment of vsOVCFs with spinal canal compromise, achieving significant vertebral height restoration and kyphotic angle reduction and leading to a significant pain relief and improvement in function.
